# Advancement toward Polymer Electrolyte Membrane Fuel Cells at Elevated Temperatures

**DOI:** 10.34133/2020/9089405

**Published:** 2020-06-08

**Authors:** Jin Zhang, David Aili, Shanfu Lu, Qingfeng Li, San Ping Jiang

**Affiliations:** ^1^Beijing Key Laboratory of Bio-Inspired Energy Materials and Devices & School of Space and Environment, Beihang University, Beijing 100191, China; ^2^Department of Energy Conversion and Storage, Technical University of Denmark, Fysikvej 310, 2800 Lyngby, Denmark; ^3^Fuels and Energy Technology Institute & WA School of Mines: Minerals, Energy and Chemical Engineering, Curtin University, WA6102, Perth, Australia

## Abstract

Elevation of operational temperatures of polymer electrolyte membrane fuel cells (PEMFCs) has been demonstrated with phosphoric acid-doped polybenzimidazole (PA/PBI) membranes. The technical perspective of the technology is simplified construction and operation with possible integration with, e.g., methanol reformers. Toward this target, significant efforts have been made to develop acid-base polymer membranes, inorganic proton conductors, and organic-inorganic composite materials. This report is devoted to updating the recent progress of the development particularly of acid-doped PBI, phosphate-based solid inorganic proton conductors, and their composite electrolytes. Long-term stability of PBI membranes has been well documented, however, at typical temperatures of 160°C. Inorganic proton-conducting materials, e.g., alkali metal dihydrogen phosphates, heteropolyacids, tetravalent metal pyrophosphates, and phosphosilicates, exhibit significant proton conductivity at temperatures of up to 300°C but have so far found limited applications in the form of thin films. Composite membranes of PBI and phosphates, particularly *in situ* formed phosphosilicates in the polymer matrix, showed exceptionally stable conductivity at temperatures well above 200°C. Fuel cell tests at up to 260°C are reported operational with good tolerance of up to 16% CO in hydrogen, fast kinetics for direct methanol oxidation, and feasibility of nonprecious metal catalysts. The prospect and future exploration of new proton conductors based on phosphate immobilization and fuel cell technologies at temperatures above 200°C are discussed.

## 1. Introduction

**Figure 1:**
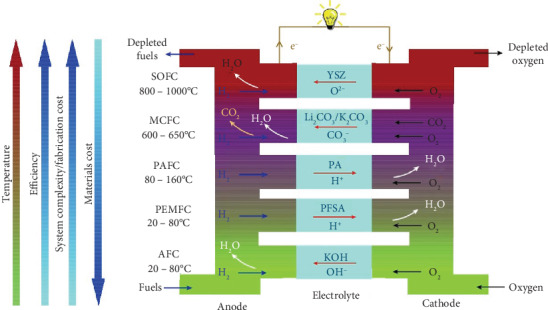
Types of fuel cells and relative trend in relationship between the materials cost, system complexity/fabrication cost, efficiency, and operational temperature of various fuel cells [1].

Energy and environment are of a global concern, which has stimulated worldwide research on clean, efficient, and sustainable energy technologies. Hydrogen and fuel cells are recognized as possible solutions in this context. Fuel cells convert chemical energy of fuels into electricity with inherently high energy efficiency. So far, the most developed fuel cell types are the low temperature (80-200°C) polymer electrolyte membrane and phosphoric acid fuel cells (PEMFCs and PAFCs) and the high temperature solid oxide and molten carbonate fuel cells (SOFCs and MCFCs) (see Figure 1) [1]. PEMFCs typically operating at below 80°C show high power output and have therefore attracted great attention for automobile, stationary as well as portable applications [2, 3]. Despite the high mutuality of the PEMFC technology, establishment of a hydrogen infrastructure, i.e., the storage, transportation, and distribution of pure H_2_ is the main barrier to large-scale commercialization [4, 5].

Compared to H_2_ (1.9 MJ L^−1^ at 20 MPa), liquid alcohol fuels, e.g., methanol (CH_3_OH, 17.28 MJ L^−1^), have a high energy density and flexible resources and are easy in storage and distribution [6]. However, wide applications of PEMFCs based on direct oxidation of alcohol fuels are retarded by the low catalyst activity at low temperatures [3, 7]. An alternative solution is to generate hydrogen by an on-board reformer. The reformate hydrogen is, however, containing CO_2_ and CO, the latter being a strong poison to the anode catalyst. For PEMFCs operating at below 80°C, the catalyst tolerance to CO is very poor, below 10-20 ppm [8] and careful removal of CO after reforming is demanded, which increases the complexity of the fuel processing systems. The CO poisoning effect is temperature dependent. A variant of PEMFCs operating at above 100°C is developed based on acid-doped polybenzimidazole (PBI) membranes [9, 10], called high temperature PEMFCs or HT-PEMFCs. A significantly higher CO tolerance, as high as 30,000 ppm, has been demonstrated at temperatures of 150-200°C [11, 12]. Power units and modules ranging from 1.4 kW to 75 kW are commercially available, which are constructed with HT-PEM stacks equipped with simple, external methanol reformers. The fuel cell reaction is exothermic, and the heat produced can be used for preheating. This partial thermal integration of the fuel cell stack and methanol reformer improves the overall efficiency.

Complete integration of the fuel cell stack with a reformer is desirable. The reforming is endothermic, while the fuel cell stack releases about 3 times more heat than the reformer needs. Even an effective cooling is an issue for fuel cell stacks. The same argument applies for the water supply. Integration is also expected to lead to simple and compact construction of the system and therefore fast load and thermal response. Use of the fuel cell waste heat for reforming is, however, only possible when the temperatures of the reformer and fuel cell stack are matching. The methanol steam reforming (MSR) requires to operate at temperatures over, say, 220°C in order to reach sufficient kinetics, which is a key to high reforming rate of methanol and production of H_2_-rich gas [13]. Thus, to develop the integrated MSR-PEMFC power systems, the operation temperature of PEMFCs needs to be in a range of 240-300°C to achieve high system efficiency and compact design.

Substantial progresses and achievements have been obtained in the last two decades to approach an intermediate temperature of operation, which in the present review is defined as a gap between 200 and 300°C. This is a relatively low temperature range compared with the SOFC technology, although attempts to reduce the operating temperature by development of oxide-based proton conductors have been explored. These efforts are however not included in the review, and the reader is referred to recent reviews [14, 15]. From the PEMFC side, great efforts have been made and significant advancement has been achieved toward the intermediate temperature. Successful protocols include developments of acid-doped basic polymers and inorganic proton conductor [16–18]. Polybenzimidazole- (PBI-) based membrane electrolytes and fuel cell technologies have been well reviewed and recently updated [19, 20]. Inorganic proton conductors based on phosphates and solid acids have also been explored as electrolytes for fuel cells [21]. Preparation of inorganic-organic composites is an effective way to advanced membrane materials [22, 23], particularly to reduce methanol crossover [24]. The subject has been very recently reviewed [25].

This review is devoted to a summary of the state-of-the-art acid-doped PBI and solid inorganic proton conductors. The main focus is placed on updating of the advancement of inorganic/organic composite membranes and proton carriers particularly for operation at elevated temperatures, e.g., 200°C or higher, in order to enable a novel integrated power systems, as outlined recently [26]. Prospect and future direction in the development of new proton carrier systems at elevated temperatures are discussed.

## 2. Phosphoric Acid-Doped Polybenzimidazole Membranes

The early success with phosphoric acid-doped PBI (PA/PBI) membranes as high temperature polymer electrolytes has triggered tremendous research and development of new PBI chemistries during the last two decades. An overview of the structural scope of PBI in this context from a polymer chemistry perspective can be found in the excellent reviews by, e.g., Asensio et al. [27] and Quartarone and Mustarelli [28].

**Figure 2:**
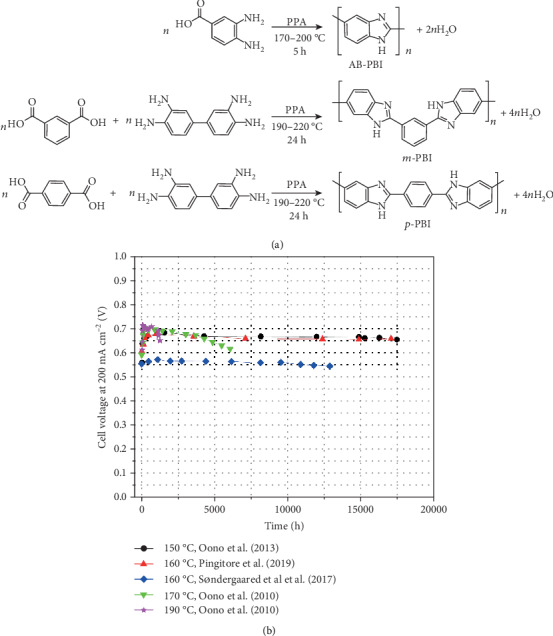
(a) Homogenous solution polymerization of common PBI derivatives. (b) Summary of HT-PEM fuel cell durability data recorded at 150-160°C and 200 mA cm^−2^ reported by Oono et al. [55, 56], Pingitore et al. [57], and Sondergaard et al. [58]. The reader is referred to the cited references for further details about the MEA components and test conditions.

In the scientific community, PBI derivatives are typically produced by homogenous solution polymerization using polyphosphoric acid (PPA) as the polycondensation solvent. The most structurally simple derivative, AB-PBI, is obtained from 3,4-diaminobenzoic acid, as shown in Figure 2(a) [29]. The most widely used PBI derivative is, however, based on poly(2,2′-(*m*-phenylene)-5,5′-bibenzimidazole) (*m*-PBI). The main reason is that *m*-PBI, in contrast to AB-PBI, can be dissolved in common organic solvents used for polymer processing. Membranes are typically cast from polar aprotic organic solvents, such as *N*,*N*-dimethylacetamide, and thereafter doped with PA by equilibrating the membrane in aqueous PA solutions. Equilibrating in 85% H_3_PO_4_ at room temperature typically results in an acid uptake corresponding to ~10-12 H_3_PO_4_molecules per polymer repeat unit [30]. Compared with the traditional batch method, a roll-to-roll process was found to increase the *m*-PBI membrane fabrication speed 100 times, yielding 250 mm wide and 40 *μ*m thick *m*-PBI membranes at 12 m h^−1^ with good thickness homogeneity [31].

Like AB-PBI, the *p*-PBI analogue is not soluble in organic solvents but can be processed directly from the crude PPA polycondensation solution to give free-standing and mechanically strong membranes with PA contents corresponding to 30-40 H_3_PO_4_moleculaes per polymer repeat unit, called the PPA process [32]. Since it involves fewer process steps than the organic solvent route, it is suitable for upscaling and production on an industrial scale [33]. The membranes produced according to the PPA process differ morphologically from that prepared by solution casting from organic solvents and even show higher conductivity at similar PA loadings [34].

It is a general understanding that the proton conductivity of PA/PBI membranes increases with PA doping. Under dry conditions at a typical fuel cell operating temperature of 160°C, the ion conductivity reaches around 50, 150, and 240 mS cm^−1^ at PA contents corresponding to about 6, 11, and 30 H_3_PO_4_ per polymer repeat unit, respectively [35, 36]. The conductivity increases with increasing temperature but tends to level off or decline at temperatures above 160°C due to condensation of the PA to pyrophosphoric acid and higher oligomers with lower conductivity [37]. Active humidification, on the other hand, suppresses the dehydration and results in a significant conductivity enhancement. For example, for an *m*-PBI membrane with a PA loading corresponding to around 11 H_3_PO_4_ per polymer repeat unit, the conductivity under dry conditions was found to increase from 40 to 70 mS cm^−1^ with the increase in temperature from 120 to 180°C, while it increased from 120 to 140 mS cm^−1^ under fully humidified conditions [38].

In addition to supporting proton conductivity, the PA within the membrane also exerts a strong plasticizing effect, and the PA loading must therefore be balanced based on high conductivity on one hand and mechanical robustness on the other. The intermolecular hydrogen bonding between the benzimidazole groups in *m*-PBI results in a relatively close polymer chain packing and a density of 1.33 g cm^−3^ [39]. At intermediate molecular weights, the room temperature elastic modulus of *m*-PBI is around 3-4 GPa, while the tensile strength at break typically ranges from 100 to 160 MPa [36, 38]. After doping with phosphoric acid, an *m*-PBI membrane of comparable molecular weight with a PA loading corresponding to about 11 H_3_PO_4_ per polymer repeat unit showed an elastic modulus of 48 MPa and a tensile stress at break of 15 MPa [38]. By further increasing the temperature towards a practical fuel cell operational temperature of 130°C, the elastic modulus and engineering tensile stress at break further dropped to 1.3 and 0.4 MPa, respectively.

Different strategies have been explored to mitigate the dramatic softening of PBI membranes in the PA-doped stage and to improve the mechanical robustness at high PA contents. The most straightforward approach is to increase the linear molecular weight of the *m*-PBI used for the membrane fabrication [36]. Altering the linear structure, for example, by employing *p*-PBI or dihydroxy functionalized PBI, is another effective approach [40]. Covalent crosslinking by *N*-coupling of the benzimidazole groups by cocasting the *m*-PBI together with a bifunctional electrophile, such as *α*,*α*′-dibromo-*p*-xylene, bis(chloromethyl)arenes, 1,3,5-tris(bromomethyl)benzene, dichloromethyl phosphonic acid, divinylsulfone, or silanes has been extensively explored. Interpenetrating networks or polymer blends with polymeric crosslinkers have also been investigated in this context, based on structures containing halomethyl groups such as halomethylated polysulfone [41], poly(vinylbenzyl chloride) [42], or bromomethylated poly(aryl ether ketone) [43], which react in a similar fashion as the bifunctional electrophiles provided that a homogenous blend can be obtained. Ionic crosslinking [44] and mechanical reinforcement with a stable inert phase [45] have also been successfully demonstrated towards enhancing the mechanical robustness at high PA loadings.

On a fundamental level, the success with PA/PBI membranes in high temperature PEMFCs is attributed mainly to the combination of low vapor pressure and high intrinsic conductivity of PA at low water activities, and the high degree of Grotthuss-type structure diffusion proton conductivity mechanism [46, 47]. In 99% H_3_PO_4_, the proton transference number is as high as 0.975 [48]. However, the contribution of vehicular ion conduction increases at higher water contents due to the promoted acid dissociation and also increases upon further drying due to the formation of condensed phosphoric oxoacid charge carriers, such as H_2_P_2_O_7_^2-^ or H_3_P_3_O_10_^2-^ [49]. The contribution from the vehicular proton conductivity mechanism is problematic from a fuel cell operation and durability point of view. Ultimately, the parasitic migration of phosphoric oxoacid species due to electroosmotic drag would result in the development of a PA concentration gradient across the membrane and ultimately PA flooding at the anode and PA depletion at the cathode. The redistribution and migration of PA have been confirmed by *in-operando* X-ray tomography imaging [50, 51] as well as by resistance measurements of different layers in a segmented membrane electrode assembly (MEA) [52]. The redistribution of the PA may be a trigger for PA loss, which is the main factor causing the degradation at temperatures beyond 180°C or high current densities of 600-800 mA cm^−2^ [53]. Yu et al. [54] indicated that the PA leaching rate at the cathode has been found to be about an order of magnitude higher when the fuel cell operating temperature increased from 160°C to 190°C. Crosslinking or creep resistance improvements of the membrane have been proven to be one of the most effective degradation mitigation strategies, and cells constructed based on such membranes have repeatedly been demonstrated to operate for up to 18000 h with average cell voltage degradation rates as low as 1.8 *μ*V h^−1^, as summarized in Figure 2(b) based on durability data reported in the literature [55–58]. It should be remarked that the cells showing the lowest average degradation rates were operated under mild conditions to suppress different modes of degradation. First, the relatively low temperature of 150-160°C results in low PA loss and prevents the acid from condensing to higher oligomers. Furthermore, the relatively low current load of 200 mA cm^−2^ prevents PA redistribution due to migration and the relatively low cell voltage assures a low rate of catalyst and electrode degradation due to, e.g., platinum dissolution and carbon corrosion.

The underlying reason for the improved stability of the crosslinked and creep-resistant membranes remains to be explained. A plausible explanation is that the enhanced dimensional stability of the crosslinked or creep resistant membranes protects the membrane from the sudden mechanical collapse, which was observed by Oono et al. [55, 59] and identified as the main reason for the sudden performance decay after test at 150°C and 200 mA cm^−2^ for 14000 h. Furthermore, dimensional instability of the membrane could be a potential trigger for PA loss, which is a predominating degradation mechanism at high current loads and at temperatures above 160°C [53, 60]. Interestingly, membranes based on alternative phosphoric acid doped pyridine containing aromatic polyethers allow for stable fuel cell performance at considerably higher temperatures than PBI-based cells (up to 220°C), which may be related to better structural stability of the membrane [61].

Significant industrial efforts have been made to produce the materials and technology based on polybenzimidazole membranes. Membranes and MEAs are commercially available from FumaTech, Danish Power Systems (Dapozol®), Advent (TPS®), and BASF (Celtec®). The stacks and methanol-fueled power units are supplied by SerEnergy, Advent, and Siqens and probably soon by the newly established Blue World Technologies, among others.

## 3. Phosphate-Based Solid Inorganic Proton Conductors

Solid inorganic proton conductors have been synthesized for fuel cell applications at elevated temperatures of 100-300°C. There are a large number of publications in this area [62–64]. Among others, trivalent metal phosphates (e.g., boron) and rare earth metal (e.g., La and Gd) phosphates are also reported [65]. However, their conductivities are not sufficiently high for practical applications. Protic ionic liquids have also been of interest but have not yet been successfully implemented in fuel cells. In the following, the discussion is limited to alkali metal or ammonium dihydrogen phosphates, heteropolyacids, tetravalent metal pyrophosphates, and phosphosilicate or silicophosphates.

### 3.1. Dihydrogen Phosphates

Solid acids, particularly alkali metal or ammonia dihydrogen phosphates, e.g., cesium dihydrogen phosphate (CsH_2_PO_4_, CHP), are well-known proton conductors. This type of acidic salts is characterized by a superprotonic phase-transition temperature at ~230°C [66]. A dynamic disordered hydrogen bond network is developed above this temperature, leading to a jump in the proton conductivity by 4-5 orders of magnitude, i.e., 8.5 × 10^−6^ at 223°C to 1.8 × 10^−2^ S cm^−1^ at 233°C [21]. The superprotonic phase is stable at up to 250°C, above which the dehydration takes place forming polymerized products such as CsH_2_P_2_O_7_ or eventually CsPO_3_ unless a humid atmosphere containing up to 30 mol% water vapor is provided [67].

The very narrow temperature range of the superprotonic phase is a challenge for any technological applications of the pure CsH_2_PO_4_. To solve this problem, heterogeneous doping of the acidic salt has been explored. To extend the temperature to a high limit, composites of the CHP with oxides [67–69] or phosphates [70, 71] have been explored. For practical applications, another challenge is to formulate an electrolyte layer with sufficent mechanical strength in a process that is scalable.

### 3.2. Heteropolyacids

**Figure 3:**
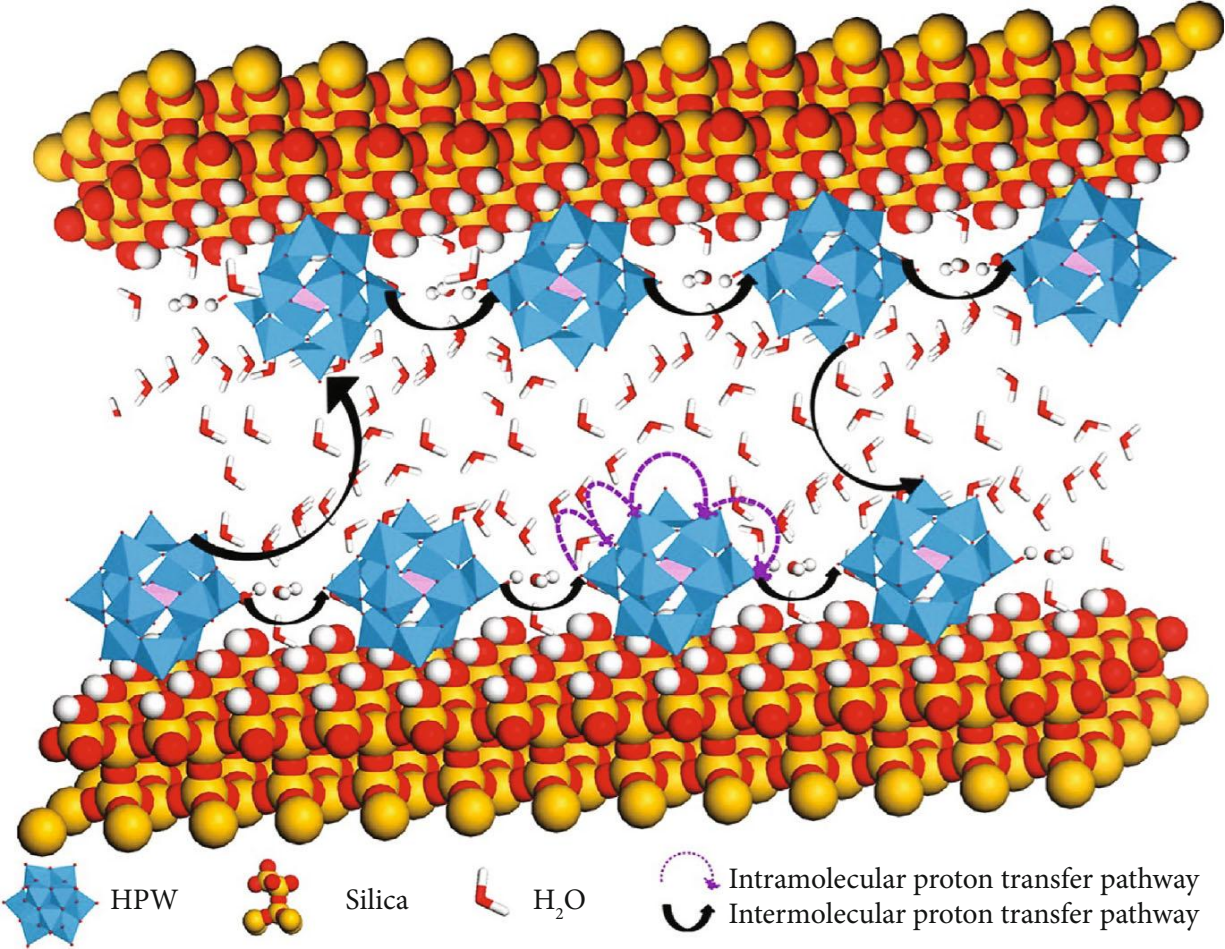
The proposed proton transportation on PWA-*meso*-silica in two ways. One is proton hopping on an isolated phosphotungstic acid, briefly as the intramolecular proton transfer pathway. The other is intermolecular proton transfer pathway, in which the proton-exchange process is composed of a series of “hops” among HPW or PWA molecules and water molecules along the hydrogen bond [76].

Heteropolyacids (HPA), e.g., phosphomolybdic acid (PMA), phosphotungstic acid (PWA), and silicotungstic acid (SiWA), are known to have high intrinsic proton conductivity of up to 0.18 S cm^−1^ at room temperature with full hydration [18, 72]. Nevertheless, heteropolyacids are soluble to water, leading to failure of proton conductor membranes made of pure heteropolyacids. A strategy was to support the heteropolyacids on mesoporous silica. A significant work has been done by immobilization of the heteropolyacids in porous silica (e.g., PWA) via impregnation [73] or self-assembly [74, 75] (PWA-*meso*-silica). The PWA was found to be confined and immobilized in the mesoporous silica, which significantly increased the water resistance compared to the simple blending PWA and mesoporous silica. The negatively charged ions, PW_12_O_40_^−3^, are anchored inside the highly ordered mesoporous silica channels of the membrane. The distances between PWA nanoclusters depend on the loading of PWA, and with the increase of content of PWA, the average distance between PWAs decreases. This leads to a reduced energy barrier of proton migration and thus increased proton conductivity. The conductivity depends on the rates of two proton transfer pathways, as depicted in Figure 3 [76]: the intramolecular proton transfer, in which the proton hopping occurs on an isolated PWA, and the intermolecular proton transfer, in which proton hopping occurs between PWAs via the water-assisted hydrogen bond. The PWA-*meso*-silica shows a high proton conductivity of 3.4 × 10^−2^ S cm^−1^ at 200°C and good fuel cell performance [77, 78]. In addition, the PWA-*meso*-silica membrane was also assembled into fuel cell stack (10 cells), producing a peak power of 74 W (372 mW cm^−2^) at 150°C and 100% relative humidity (RH) fed by H_2_ and O_2_ [79].

Nevertheless, the proton conductivity of heteropolyacids is found to be highly dependent on the presence of water [72]. From a technological point of view, it means that active humidification of the feed gases is needed, which makes the fuel cell construction and operation complex.

### 3.3. Tetravalent Metal Pyrophosphates

**Figure 4:**
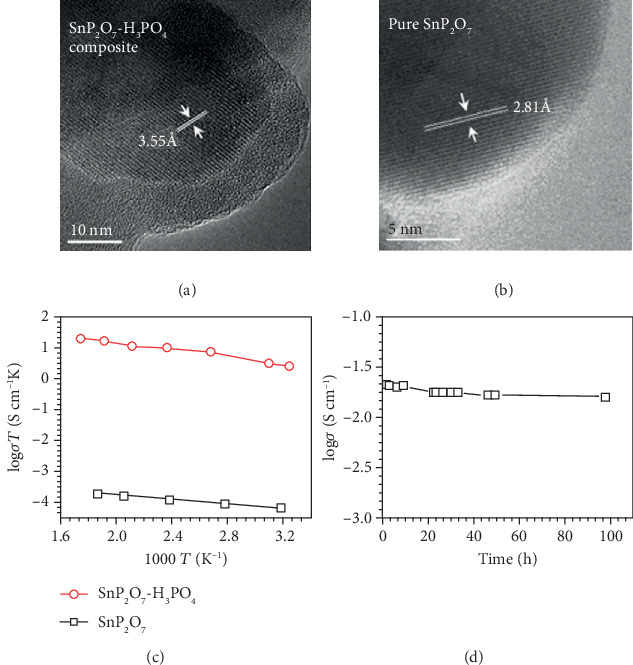
TEM images of (a) SnP_2_O_7_-H_3_PO_4_ and (b) pure SnP_2_O_7_. (c) Temperature dependence of proton conductivity for SnP_2_O_7_-H_3_PO_4_ and pure SnP_2_O_7_ in air. (d) Proton conductivity of SnP_2_O_7_-H_3_PO_4_ at 250°C in air as a function of time [87].

Metal pyrophosphate has been recognized as one of the promising high temperature proton conductors due to their high proton conductivity at 200-300°C under dry conditions [80, 81]. There are a few good reviews on pyrophosphates [82, 83]. The crystalline SnP_2_O_7_ shows low proton conductivity (10^−9^-10^−5^ S cm^−1^) [84] at 250°C when it was obtained at high temperature over 1050°C. By using lower sintering temperatures of 650°C and high *P*/*M* ratio (>2.4), the proton conductivity can reach as high as 10^−2^ S cm^−1^ at 250°C under dry conditions [85]. The conductivity difference is due to the fact that the SnP_2_O_7_ sintered at low temperature has a core-shell structure [86]. Xu et al. [87] discovered two layers of a crystalline SnP_2_O_7_ core and an amorphous shell (denoted as SnP_2_O_7_-H_3_PO_4_) by transmission electron microscopy (TEM) when SnO_2_ and H_3_PO_4_ were sintered at 350°C (Figure 4(a)). On the contrary, the amorphous layer is absent in the pure crystalline SnP_2_O_7_ particles (Figure 4(b)). The amorphous shell may contain excess phosphoric acid or HPO_3_ derived from phosphoric acid that decomposes during sintering or phosphoric oxoacid species formed by hydration of amorphous P_m_O_n_ [87, 88]. For instance, residual PA had been confirmed by ^31^P NMR in the core-shell SnP_2_O_7_-H_3_PO_4_ with a P : Sn ratio of 4 : 1. In addition, the SnP_2_O_7_-H_3_PO_4_ reached high proton conductivity up to 3.5 × 10^−2^ S cm^−1^ at 300°C in air (Figure 4(c)) and showed stable proton conductivity at 250°C for 100 h (Figure 4(d)). Consequently, it is reasonable to conclude that the high proton conductivity of the core-shell pyrophosphates is due to the acidic amorphous shell.

Doping with trivalent metal ions (e.g. Al^3+^, Ga^3+^, Gd^3+^, or In^3+^) is of special importance to improve conductivity of the pyrophosphate proton conductors [86]. The incorporation of dopants induces point defects to the pyrophosphate crystal structure. The point defects interacted with the protons from water and then increased the proton concentration of the materials. Nevertheless, the conductivity of the pure crystalline In^3+^-doped SnP_2_O_7_ (e.g., Sn_0.9_In_0.1_P_2_O_7_) is only 10^−6^ S cm^−1^ at 900°C [89]. Heo et al. reported a high proton conductivity of 4.0 × 10^−2^ S cm^−1^ at 200°C and 0% RH for Sn_0.9_In_0.1_P_2_O_7_ and a peak power density (PPD) of 284 mW cm^−1^ in H_2_/air on a PEMFC based on the membrane at the same test conditions [90]. The high proton conductivity of Sn_0.9_In_0.1_P_2_O_7_ is believed to result from the trace amount of an amorphous secondary phase smothered on the surface of the crystalline Sn_0.9_In_0.1_P_2_O_7_ [91]. Besides trivalent ions, bivalent dopants including Mg^2+^ and Zn^2+^ have also been employed to substitute the tetravalent ions. The optimized composition of Sn_0.9_Mg_0.1_P_2_O_7_ shows lower proton conductivity than that of Sn_0.9_In_0.1_P_2_O_7_ at the identical conditions due to the low proton mobility of Mg^2+^ with high electrostatic restriction against proton transport [64]. Nevertheless, basic MgO reduced the acidity of the SnP_2_O_7_ due to neutralization, which enhances the stability of the Pt catalyst and the carbon support that were in contact with the Sn_0.9_Mg_0.1_P_2_O_7_ electrolyte [64]. It should be noted that the particle size and morphology of the metal ions of substituted pyrophosphate also significantly affect the proton conductivity of the materials. The critical challenge for technological applications of these proton conductors is to fabricate mechanically strong and gas tight electrolyte films as ceramic-like sintering processes produce porous structures of phosphates.

### 3.4. Phosphosilicate

There are a few reports on the preparation of phosphosilicate from sol-gel reaction [92, 93] or phosphonic acid functionalized silicas [94]. Tadanaga et al. [95] synthesized phosphosilicate Si_5_O(PO_4_)_6_ by blending the tetraethoxysilane (TEOS) and H_3_PO_4_ at 150°C with proton conductivity of 2.5 × 10^−3^ S cm^−1^ at 180°C under more than 0.4% RH. Furthermore, when TEOS and PA were reacted in a hydrothermal reactor at 150°C, a monolithic phosphosilicate membrane was obtained [96]. On the contrary, the silica membrane soaked with PA solution showed rapid proton conductivity decay from 10^−2^ to 10^−6^ S cm^−1^ within 400 min under the same test conditions.

Recently, Ansari et al. [97] prepared phosphosilicate with a novel amorphous form of zeolitic silica, denoted as SiPOH, from reaction of PA with SiCl_4_ at 270°C. SiPOH has a likely structure of Si(PO_4_H)_2_ as clarified by the ^29^Si NMR. In addition, the SiPOH contains a six-coordinate silicon state and the Si to P ratio is 1 : 4 from the ICP analysis. SiPOH tends to confine PA molecules in the nanopore of the materials and immobilize PA with an equilibrium of H_4_P_2_O_7_ up to 250°C [97]. This leads to a proton conductivity of SiPOH compared with that of the H_3_PO_4_ at above 150°C. When reinforced with glass fibers, a flexible, inorganic membrane was obtained which has been assembled into a fuel cell performing at temperatures of at least up to 226°C [98].

## 4. Polybenzimidazole-Phosphate Composite Membranes

As described above, PBI has emerged as the most common HT-PEM and the research in this area has largely focused on balancing the proton conductivity versus the mechanical robustness. The incorporation of inorganic additives has been widely studied in this connection, aiming at increasing the mechanical robustness and thermal stability of the PA/PBI membrane. The types of inorganic fillers include metal oxides (SiO_2_, TiO_2_, Fe_2_TiO_5_, etc.), solid acids (heteropolyacids, zirconium phosphate, and cesium salts), carbon-based materials, metal organic frameworks, and clays (aluminum silicates) [25]. In general, there are two strategies to introduce the inorganic fillers into the polymer: one is by dispersing the filler in a polymer solution and cocasting a membrane while the other by *in-situ* formation of the inorganic component inside the polymer from precursors introduced. In the following, the discussion is selectively devoted to those that are shown or have potential to extend the operational temperatures close to or higher than 200°C.

### 4.1. PBI/Dihydrogen Phosphate Composite Membranes

Generally, the solid inorganic materials are incorporated to a polymer matrix to form the inorganic-organic composite membranes. He et al. [99] revealed that the proton conductivity of a PA-doped PBI/Zr(HPO_4_)_2_ membrane at 200°C and 5% RH was 50% higher than that of the pristine PA/PBI membrane with the same PA content (5.6 PA per polymer repeat unit) tested under similar conditions. The Zr(HPO_4_)_2_ was also found to enhance the PA retention capability of the PA/PBI membrane in humidified conditions.

Compared to inorganic phosphates, organic-inorganic metal sulfophenyl phosphate (SPP) shows higher compatibility with the polymer matrix. Controlled membrane formation and good morphological control are achieved for the polymer/metal sulfophenyl phosphates composite membranes. Sun et al. [100, 101] have conducted comprehensive research on the application of PA/PBI membrane with the addition of CeSPP and FeSPP. The proton conductivity of the PBI/CeSPP composite membrane with 25 wt% CeSPP reached up to 1.1 × 10^−1^ S cm^−1^ at 180°C and 100% RH. Nevertheless, the proton conductivity of metal sulfophenyl phosphate is highly dependent on the RH, resulting in rapid proton conductivity decay at low RH for the composite membrane [101]. In addition, the mechanical strength of the PBI/SPP composite could be further increased by introduction of glass fibers as reinforcement [100].

### 4.2. PBI/Pyrophosphate Composite Membranes

Wu et al. [102] incorporated Sb_0.2_Sn_0.8_P_2_O_7_ nanoparticles into the PBI matrix with uniform distribution at a loading of 20 wt%. However, the PBI/inorganic composite membranes were prone to break into pieces at inorganic particle contents above 40 wt% [103]. This may be due to the large aggregate of the inorganic particles, which substantially reduces the mechanical property of the PBI membrane via the cast method. When Sn_0.95_Al_0.05_P_2_O_7_ was incorporated to the PBI matrix by the *in situ* reaction of SnO_2_ and Al(OH)_3_ with phosphoric acid at 250°C, the loading of SAPO particles reached up to 43.3%, while the SAPO membrane showed a high proton conductivity of 0.1 S cm^−1^ at 300°C [104]. Recently, an outstanding pyrophosphate composite membrane Sn_0.95_Al_0.05_P_2_O_7_ (SAPO) has been fabricated by Lee et al. [105] via the casting of SAPO and perfluorinated Nafion® ionomer mixture, while the membrane cells achieved the PPD of 840 mW cm^−2^ at 240°C in H_2_/O_2_. Besides the casting method, the inorganic membranes including SAPO and SnP_2_O_7_ could be pressed into pellet with binders such as polytetrafluoroethylene (PTFE) or sulfonated polystyrene-b-poly(ethylene/butylene)-b-polystyrene (sSEBS). The content of the binders for the inorganic membrane varied from 5 wt% to 20 wt% [73, 74].

The incorporation of metal pyrophosphate increases the proton conductivity of the polymer membrane at high temperature. Jin et al. [103] employed ^1^H and ^31^P MAS NMR to detect PA/PBI and PA/PBI/SAPO membranes and demonstrated that both membranes show the same ^1^H and ^31^P peaks, suggesting the same proton diffusion mechanism between SAPO and PA. However, this is contrast to the higher proton conductivity and stability of SAPO compared to PA at temperatures above 200°C [56, 87]. One possible reason is that the proton conductivity is significantly related to the distribution and microstructure of the SnP_2_O_7_ and PA phase formed. Nevertheless, the fundamental reasons for the proton diffusion mechanism of SnP_2_O_7_-based polymer electrolyte membranes are still not clear.

### 4.3. PBI/Phosphosilicate Composite Membranes

Increasing the operational temperature of the HT-PEMFC tends to decrease the durability of the cell. That is due to the dehydration and evaporation of PA as well as the degradation of the polymer at the harsh conditions. It has been revealed that the PA loss rate increases one order of magnitude from 150°C to 190°C [54]. Thereby, at a constant current density of 0.2 A cm^−2^, the durability of the fuel cell based on PA/PBI membrane decreases from ~18000 h to 1000 h with the temperature increase from 150 to 190°C [54].

**Figure 5:**
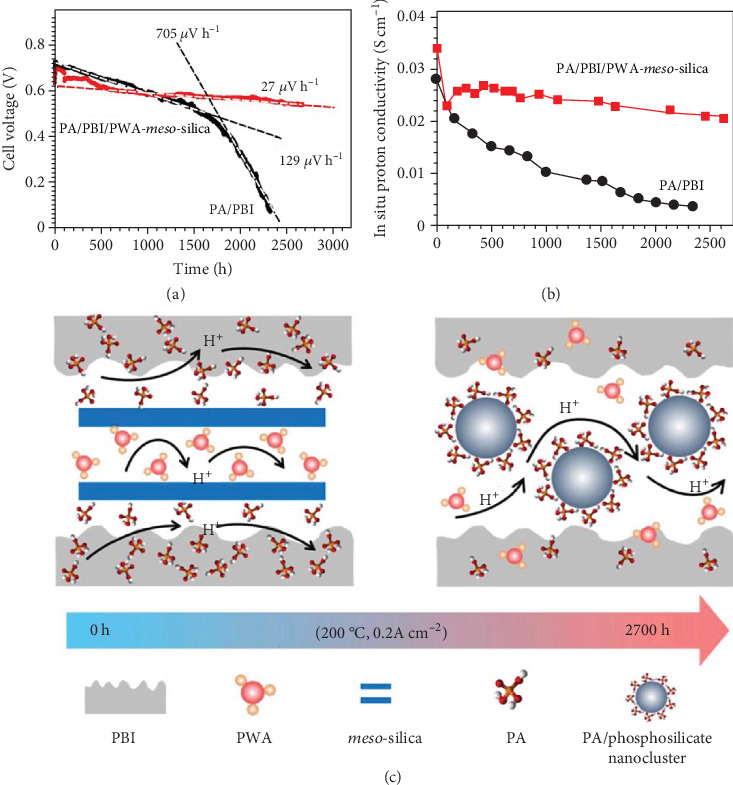
(a) Stability of PA/PBI/PWA-*meso*-silica and PA/PBI membrane cells and (b) membrane conductivity as a function of test time, measured at 200°C and 200 mAcm^−2^. (c) Scheme showing the *in situ* formation of PA/phosphosilicate nanoclusters from the interaction between the *meso*-silica and PA during the fuel cell operation conditions [106, 107].

Interaction of PA with inorganic fillers in composite membranes appears to stabilize the PA-doped polymer membranes at high temperatures. We fabricated PA-doped PWA impregnated mesoporous silica (PBI/PWA-*meso*-silica) composite membranes and observed exceptional stability of PA/PBI/PWA-*meso*-silica-based membrane cells for almost 3000 h at 200°C at a constant current density of 200 mA cm^−2^ [106]. The degradation rate of the HT-PEM fuel cell based on the composite membranes at 200°C was 27 *μ*V h^−1^ that is comparable to that of the PA/PBI fuel cell operated at 170°C [56], as shown in Figure 5(a) [106, 107]. The pristine PA/PBI membrane cell shows a sharp performance decay (129 *μ*V h^−1^) and completely fails after testing under identical conditions for 1500 h; then the fuel cell operation was ceased after a rapid voltage drop of 705 *μ*V h^−1^ for 800 h. The good stability of the PA/PBI/PWA-*meso*-silica composite membrane cell expressed as membrane conductivity is shown in Figure 5(b). As schematically illustrated in Figure 5(c), the high proton conductivity is most likely due to the in situ formation of a PA/phosphosilicate nanocluster phase within the PBI polymer matrix [107]. The nanocluster phase substantially stabilizes the phosphoric acid phase for the proton transfer at elevated temperatures.

**Figure 6:**
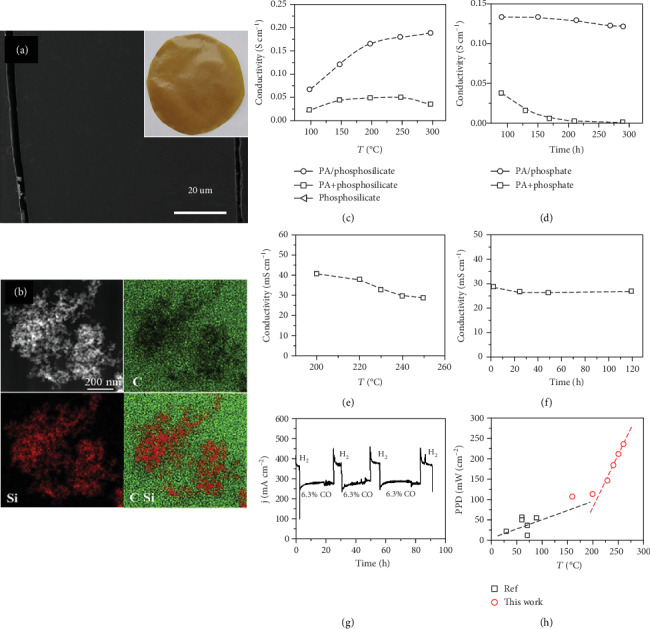
(a) Cross-sectional microstructure of the PBI/SiO_2_ membrane. The insert is the optical image of the membrane. (b) STEM-EDS element mapping of the PBI/SiO_2_ membrane. (c, d) Conductivity plots of PA/phosphosilicate, PA+phosphosilicate, and phosphosilicate composites. (e, f) Proton conductivity and stability of pretreated PA/PBI/SiO_2_ membrane. Stability data in (d) and (f) were measured at 250°C. (g) CO tolerance of PA/PBI/SiO_2_ membrane cells, measured at a cell voltage of 0.6 V in pure H_2_ and 6.3% CO in H_2_ fuel at 240°C. Air was used as an oxidant [108]. (h) PPD plots of DMFCs with PtRu/C or PtRu/CNT catalyst loading of 1.0-2.0 mg cm^−2^. Empty square symbols represent references cited in [110].

Further studies indicate that reaction between silica nanoparticles and PA will lead to the formation of “flower-like and plated” phosphosilicate particles that are attached by a layer of phosphorous species (see Figures 6(a) and 6(b)) [108]. The presence of the phosphorous species layers is important for the high proton conductivity and stability of the PA/PBI/SiO_2_ composite membranes at high temperatures. The conductivity of the phosphosilicate phase formed in PA *in situ* at 250°C for 24 h (PA/phosphosilicate) increases with the temperature, reaching a proton conductivity as high as 1.9 × 10^−1^ S cm^−1^ at 300°C under anhydrous conditions, while the conductivity of the pristine phosphosilicate is very low (3.6 × 10^−3^ S cm^−1^) under the same test conditions (Figure 6(c)) [108]. The PA/phosphosilicate phase formed *in situ* shows a relatively stable conductivity at 250°C. In contrast, the proton conductivity of the mixed PA and pure phosphosilicate, e.g., PA + phosphosilicate, composite substantially drops from 3.7 × 10^−2^ to 2.2 × 10^−3^ S cm^−1^ after being tested for 50 h (Figure 6(d)). The significant drop in the conductivity is clearly caused by the PA condensation at high temperatures. This again shows that the conductivity stability of the PA/phosphosilicate phase implies the enhanced thermal stability of the PA species in the PA/phosphosilicate nanoclusters. Compared to mixed PA+phosphosilicate phase, the proton conductivity of the *in situ* formed PA/phosphosilicate phase maintains at 1.3 × 10^−2^ S cm^−1^ for over 120 h under the same conditions. The same was also observed on the PBI membrane with embedded PA-stabilized phosphosilicate nanocluster phase at 250°C (Figures 6(e) and 6(f)). The PPD of the PA/PBI/SiO_2_ membrane cell increased from 215 mW cm^−2^ to 284 mW cm^−2^ from 200°C to 240°C, and the cell shows a good stability under test conditions of 0.6 V at 240°C for 100 h [108]. This is very different from the report that the PPD of a PEMFC based on PA/PBI/Ce_0.9_Gd_0.1_P_2_O_7_ membrane decreased from 307 mW cm^−2^ to 125 mW cm^−2^ when the operating temperature of the cell increased from 160 to 250°C [109]. This shows that the thermal stability of the proton carrier plays an important role in the power output of PEMFCs at elevated temperatures.

In addition to the high PPD and stability, the fuel cell based on PA/PBI/SiO_2_ membrane with the *in situ* formed PA/phosphosilicate phase also shows an excellent CO tolerance at 240°C (Figure 6(g)) [108]. Such high tolerance toward CO would allow the direct use of H_2_-rich fuels from methanol steam reformer. The PA/PBI/SiO_2_ composite membrane was also applied for direct methanol fuel cells (DMFCs). In the case of DMFCs, the cell showed a PPD of 136 mW cm^−2^ at 260°C with Pt/C catalyst in the anode fed with 0.1 M methanol and the PPD increased significantly to 237 mW cm^−2^ when Pt/C was replaced by PtRu/C [110]. The results indicate the existence of a distinct performance transition at about 205°C for the DMFCs (see Figure 6(h)) [110]. The increase in PPD with temperature is 208 mW cm^−2^/100°C above this transition temperature, while below 205°C, the increase in power decreases to 50 mW cm^−2^/100°C. This indicates that temperature plays a significant role in the reaction kinetics of the methanol oxidation.

## 5. Prospects in the Development of PEMFCs at Elevated Temperatures

**Figure 7:**
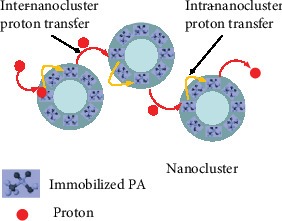
Hypothesis of inter-nanocluster and intra-nanocluster proton transfers via the immobilized phosphate nanocluster-structured proton carriers.

As shown in this review, the operational temperature of PEMFCs strongly depends on the thermal stability of the proton carriers, *i.e*., 100°C for water for Nafion-membrane-based low temperature PEMFCs and 175°C for PA for PA/PBI membrane-based HT-PEMFCs. Hence, the development of PEMFCs at elevated temperatures depends critically on the thermal stability of the proton carrier. It becomes evident that to further increase the operational temperature of PEMFCs, it is necessary to develop new proton carrier systems. From practical operation point of view, the new proton carrier must be chemically and thermally compatible with polymeric materials and thus be able to form polymer electrolyte membranes (PEMs) by conventional and scalable film fabrication techniques such as tape casting or film casting. The finding of the exceptional stability of the phosphotungstic acid-functionalized mesoporous silica (PWA-*meso*-silica)/PA/PBI composite membranes implies a potential of the *in situ* formed PA/phosphosilicate nanocluster phase as the new class of proton carriers at elevated temperatures [106, 107]. The observed high proton conductivity and stability of PA/phosphosilicate nanocluster composite demonstrate the presence of *immobilized phosphates* in the nanoclusters. This is supported by the microstructure examination that indicates the presence of an amorphous shell or layer on such *in situ* formed nanoclusters [108]. Proton transfer through such an immobilized phosphate phase in nanoclusters could proceed by a combined hopping mechanism between the nanoclusters (i.e., inter-nanoclusters) and within the nanoclusters (i.e., intra-nanoclusters), as hypothetically shown in Figure 7. On the other hand, vehicular proton conductivity mechanism would not be possible at elevated temperature from durability point of view. Such proton transfer processes do not involve “free phosphates,” as in the case of mixed PA+phosphosilicate phase and conventional PA/PBI PEMs; thus, in principle, the immobilized phosphate within the nanoclusters can conduct protons at a much higher temperature than the conventional PA/PBI PEMs. However, the grand challenges are the development and effective integration of such immobilized phosphate phases in the metal phosphate and/or pyrophosphate nanoclusters in PEMs and the fundamental understanding of the proton conduction mechanism at elevated temperatures under fuel cell operation conditions.

In fuel cell technologies, the most critical component is the electrolyte and this is particularly the case for HT-PEMFCs. The phosphate-based solid inorganic proton-conducting electrolytes demonstrate the acceptable proton conductivity at elevated temperatures above 200°C, but the low mechanical strength and brittle nature of inorganic materials make them not applicable for the PEMFC technologies with practical applications and commercial viability. Polymeric material-based electrolytes are the direction for the HT-PEMFCs. In this regard, PBI is a state-of-the-art polymer material due to its high thermal and chemical stability, as reviewed in this article. However, PBI-based polymers are relatively expensive some derivatives show processing difficulties due to poor solubility in common organic solvents. Hence, it is necessary to design and develop new and alternative polymer materials to replace PBI in HT-PEMFCs. We have shown that the polyvinylpyrrolidone- (PVP-) based blend polymer is a promising alternative for HT-PEMFCs [111, 112]. In blended membranes, PVP contains N-heterocycle, which acts as a proton acceptor, while added polymers such as polyvinylidene fluoride (PVDF) and poly(ether sulfone) (PES) improve thermal and mechanical stability in the membranes. Compared with PBI, PVP-based composite membranes have an unparalleled cost advantage due to the wide availability and cheap and easy processing capabilities.

A fundamental breakthrough in the membrane and proton carrier system with operating temperatures higher than 200°C will undoubtedly open new opportunities in the development of nonprecious metal (NPM) catalysts [113] and highly efficient and integrated MSR-PEMFC power systems [26] for practical and wide employment of fuel cell technologies in transportation, communication, and portable devices. The development of NPM catalysts will substantially reduce the cost of PEMFCs and is of great significance to the practical application and commercial viability of PEMFCs technology. An obvious benefit of the development of PEMFCs for operation at elevated temperatures is the direct utilization of liquid fuels such as methanol and substantially reduced cost associated with the storage, transport and distribution of high purity hydrogen for conventional low temperature PEMFCs. Thus, considerable R&D efforts, collaborations, and investment are required for the development of truly commercially viable and competitive PEMFC technologies for the energy convention and power source applications in the future.
